# The HCV-Melanoma Paradox: First Multi-Cohort and Molecular Net-Work Analysis Reveals Lower Incidence but Worse Outcomes—Integrating Clinical, Real-World, and In Silico Data

**DOI:** 10.3390/medicina60091531

**Published:** 2024-09-19

**Authors:** Essam Al Ageeli, Jawaher A. Abdulhakim, Mohammad H. Hussein, Maryam M. Alnoman, Samia S. Alkhalil, Peter P. Issa, Nader A. Nemr, Ahmed Abdelmaksoud, Dhaifallah A. Alenizi, Manal S. Fawzy, Eman A. Toraih

**Affiliations:** 1 Department of Basic Medical Sciences, Faculty of Medicine, Jazan University, Jazan 45141, Saudi Arabia; ealageeli@jazanu.edu.sa; 2 Department of Medical Laboratory, College of Applied Medical Sciences, Taibah University, Yanbu 46423, Saudi Arabia; jabdulhakim@taibahu.edu.sa; 3 Ochsner Clinic Foundation, New Orleans, LA 70121, USA; mohamed.hussein@ochsner.org; 4 Department of Biology, Faculty of Science, Taibah University, Yanbu 46423, Saudi Arabia; mnaaman@taibahu.edu.sa; 5 Department of Clinical Laboratory Sciences, College of Applied Medical Sciences, Shaqra University, Alquwayiyah 11961, Saudi Arabia; salkhalil@su.edu.sa; 6 School of Medicine, Louisiana State University Health Sciences Center, New Orleans, LA 70112, USA; pissa@tulane.edu; 7 Endemic and Infectious Diseases Department, Faculty of Medicine, Suez Canal University, Ismailia 41522, Egypt; nadernemr@med.suez.edu.eg; 8 Department of Internal Medicine, University of California, Riverside, CA 92521, USA; aabdelma@medsch.ucr.edu; 9 Department of Medicine, Faculty of Medicine, Northern Border University, Arar 91431, Saudi Arabia; daalenizi@nbu.edu.sa; 10 Department of Biochemistry, Faculty of Medicine, Northern Border University, Arar 1321, Saudi Arabia; 11 Center for Health Research, Northern Border University, Arar 91431, Saudi Arabia; 12 Department of Surgery, School of Medicine, Tulane University, New Orleans, LA 70112, USA; 13 Genetics Unit, Department of Histology and Cell Biology, Suez Canal University, Ismailia 41522, Egypt

**Keywords:** HCV, melanoma, IPA, molecular markers, risk factors, prognosis

## Abstract

*Background and Objectives*: The relationship between hepatitis C virus (HCV) infection and melanoma remains poorly understood. This study aimed to investigate the association between HCV and melanoma, assess outcomes in patients with both conditions, and explore potential molecular mechanisms connecting the two diseases. *Materials and Methods*: We conducted a retrospective cohort study of 142 melanoma patients, including 29 with HCV-related cirrhosis, and analyzed their clinical outcomes. For external validation, we used the TriNetX Global Collaborative Network database, comprising 219,960 propensity-matched patients per group. An in silico analysis was performed to identify the molecular pathways linking HCV and melanoma. *Results*: In the retrospective cohort, HCV-positive melanoma patients showed an increased risk of early relapse (41.4% vs. 18.6%, *p* = 0.014), recurrence (65.5% vs. 39.8%, *p* = 0.020), and mortality (65.5% vs. 23.0%, *p* < 0.001) compared to HCV-negative patients. TriNetX data analysis revealed that HCV-positive patients had a 53% lower risk of developing melanoma (RR = 0.470, 95% CI: 0.443–0.498, *p* < 0.001). However, HCV-positive melanoma patients had higher all-cause mortality (HR = 1.360, 95% CI: 1.189–1.556, *p* < 0.001). An in silico analysis identified key molecular players, including IL-6 and CTLA4, in the HCV-melanoma network. *Conclusions*: While HCV infection may be associated with a lower risk of melanoma development, HCV-positive patients who develop melanoma have poorer outcomes. The identified molecular pathways provide potential targets for future research and therapeutic interventions.

## 1. Introduction

The incidence of cutaneous melanoma varies globally among different ethnicities/geographical locations, with an estimated total number of new cases of 99,780 in both sexes in the United States in 2022 [1]. The variability in incidence suggests socio-environmental risk factors, including family history, ultraviolet-light exposure, fair skin, multiple moles, and immunosuppression [2].

While the prognosis of melanoma can vary depending on the stage at diagnosis, advancements in treatment have improved patients’ outcomes [3,4]. Several studies have reported that the outcomes of patients with melanoma could be profoundly influenced by various comorbidities, including hepatitis C virus (HCV)-related chronic liver disease [5,6,7]. Also, various concerns remain about the overall management of this particular patient subpopulation [8].

HCV is an enveloped “positive-sense single-stranded RNA (+ssRNA)” virus that increases patient morbidity and mortality [9]. It is widely known to be the leading cause of liver dysfunction due to the associated inflammation, oxidative stress, and cellular damage. Also, it can induce liver fibrosis, cirrhosis, and even hepatocellular carcinoma (HCC) [10,11]. Several studies have shown a putative association between HCV and other types of cancer, including melanoma [5,12]. This underscores the theoretical basis for exploring this potential connection and conducting a comprehensive multi-cohort and molecular network analysis to investigate the intricacies of this complex relationship.

Research exploring the involved mechanisms underlying the pathogenetic link between HCV and melanoma has unveiled intriguing insights. Notably, lichen planus was associated with both interferon and HCV, and ongoing therapeutic interventions involving interferon, often employed in diseases like melanoma, might inadvertently exacerbate existing lichen planus lesions [13,14]. Moreover, the impact of genetic variants within the interferon genes cannot be overlooked, as they not only confer resistance to pathogens but have also been implicated in influencing melanoma progression/survival in humans [15,16,17]. Some studies even report concomitant cases of lichen planus and HCV [18,19,20]. Furthermore, a compelling theory posits that the outcome of HCV is intricately shaped by the delicate interplay between host genetics, immunological responses, and viral factors, culminating in a highly personalized and multifaceted scenario [21,22]. Moreover, recent case reports/series have reported that HCV can also affect other skin conditions, such as “psoriasis, alopecia areata, vitiligo, and cutaneous lupus erythematosus” [13,23,24]. Recent studies have also shown that patients with specific gene variants may be more vulnerable to the upregulation of the inflammatory mediators that HCV can cause, leading to psoriasis [24,25].

It is worth noting that treatment for melanoma, such as immunotherapy drugs like ipilimumab or pembrolizumab, can potentially have adverse effects in patients with HCV co-infections [26]. For instance, research findings indicate that individuals diagnosed with melanoma and co-infected with HCV exhibited hepatoxicity rates similar to the established toxicity profile of ipilimumab when treated with the medication [27,28].

Considering the poorer outcomes in melanoma patients associated with other comorbidities, it has been suggested that HCV-induced liver cirrhosis could adversely influence patients with melanoma outcomes [8]. The genetic interconnection between both disorders that may have a role in this interplay requires further research to understand the complex mechanisms involved fully and to better diagnose patients with HCV and related conditions that can lead to more targeted and effective treatments for affected patients.

To the authors’ knowledge, no primary reports have investigated the relationship between melanoma and HCV-associated cirrhosis and explored the genetic interplay between these different pathologies. In this sense, our investigation seeks to fill this critical gap in the literature by exploring the clinical effect of HCV in patients with melanoma and unraveling the potential underlying molecular networks/mechanisms that interlink these two well-known pathological entities based on bioinformatics analysis. Understanding these associations within this specific context is vital for better risk stratification and management of patients with concurrent HCV infection and melanoma.

## 2. Materials and Methods

### 2.1. Retrospective Cohort Analysis

#### 2.1.1. Study Participants

Patients diagnosed with malignant melanoma and who underwent surgical resection as their primary form of treatment were included in this study. They were enrolled between 2002 and 2016 and followed up until October 2022. A total of 142 archived “formalin-fixed paraf-fin-embedded (FFPE)” specimens were included in the current study. The inclusion criteria are (a) any age and sex, (b) complete surgical resection tissues with sufficient tissues, (c) no neoadjuvant therapy administered prior to resection, (d) the presence of paired non-cancer tissues, (e) complete clinical and pathological data, and (f) no loss of follow-up. Ocular (or uveal) melanoma and concomitant cancers were excluded. Ethical approval was obtained, and written consent was waived for using archived samples.

#### 2.1.2. Clinical and Pathological Assessment

Cutaneous melanoma has been categorized as superficial spreading melanoma, nodular melanoma, acral lentiginous melanoma, and desmoplastic melanoma. The samples subjected to histopathological evaluation, including (i) the classical tumor size/site, involvement of regional lymph node (LN), and presence/absence of distant metastasis (TN staging), (ii) the measurement of tumor thickness/the degree of invasion into the skin in millimeters (i.e., Breslow thickness) (iii) the range from superficial “Level I: confined to the epidermis = melanoma in situ” to deep “Level V: invasion to the underlying structures, including the dermis, subcutaneous fat, LN, and even muscle/bone”, in which higher levels correlated to severe tumors (i.e., Clark level), (iv) the mitotic rate, either less than five and equal or more than five mitosis/high power field (HPF), and lastly, (v) the ulceration [29].

Primary outcomes, including relapse, recurrence, and mortality due to any cause, were detailed in our previous work [15]. The “Time-to-event endpoints”, such as (i) relapse-free survival, (ii) disease-free survival, and (iii) overall survival, were assessed [30].

#### 2.1.3. Statistical Analysis of the Retrospective Cohort

We utilized SPSS version 27.0 for conducting the analysis. The findings were presented as frequency/percentage or mean/standard deviation (SD). Fisher’s exact, Student’s t, and Mann–Whitney U tests were employed to evaluate the statistical significance. A *p*-value of less than 0.05 was considered as indicative of significance. The correlation between HCV and clinical/pathological and survival data was assessed. Different risk factors were evaluated using “Kaplan-Meier analysis” and “Cox proportional hazard regression”.

### 2.2. External Validation Using TriNetX Database

#### 2.2.1. Data Source

We used the TriNetX research network, a global federated health research network providing access to electronic medical records from over 150 million patients across 127 healthcare organizations. The database contains de-identified patient data, including demographics, diagnoses, procedures, medications, and laboratory values.

#### 2.2.2. Study Population

The study population was defined based on hepatitis C virus (HCV) antibody test results in serum or plasma by immunoassay, categorizing patients into HCV infection and no-infection groups, as detailed in Supplementary Table S1. The exclusion criteria included prior primary malignancies. Subsequently, patients with both melanoma and HCV infection, and a minimum 1-year follow-up, were identified and matched with melanoma patients without HCV infection.

#### 2.2.3. Propensity Scores Matching Analysis

To mitigate potential confounding factors, a 1:1 nearest neighbor propensity score matching was performed. The matching criteria included age, sex, race, body mass index (ICD-10-CM: Z68), nicotine dependence (ICD-10-CM: F17), alcohol-related disorders (ICD-10-CM: F10), and comorbidities such as hypertension (ICD-10-CM: I10–I1A), metabolic disorders (ICD-10-CM: E70–E88), and diabetes mellitus (ICD-10-CM: E08–E13). A caliper width of 0.2 of the standard deviation of the logit of the propensity score was applied.

#### 2.2.4. Outcomes

The primary outcome was the risk of melanoma development in HCV-positive patients compared to HCV-negative patients. Subsequently, for patients with both melanoma and HCV infection, the risks of recurrence after six months and all-cause mortality were assessed.

#### 2.2.5. Statistical Analysis of the TriNetX Database Cohort

All analyses were performed using the TriNetX analytics platform. Comparisons between groups before and after matching were conducted using two-sided independent *t*-tests for continuous variables and chi-square tests for categorical variables. The results were presented as percentages for categorical variables and mean ± standard deviation for continuous variables. A *p*-value < 0.05 was considered statistically significant. For the risk of melanoma in HCV patients, we calculated the risk ratio with 95% confidence intervals. Follow-up times were reported as mean ± standard deviation and median with interquartile range (IQR). In analyzing outcomes for melanoma patients, we used a Kaplan–Meier survival analysis and Cox proportional hazards regression to compare recurrence and mortality between HCV-positive and HCV-negative melanoma patients. Hazard ratios with 95% confidence intervals were calculated for recurrence after six months and all-cause mortality. The log-rank test was employed to assess the overall survival differences between groups.

### 2.3. In Silico Data Analysis

#### Exploring Putative Mechanisms Connecting HCV and Melanoma

For step 1, constructing connecting nodes between HCV and melanoma, ingenuity knowledge-based direct and indirect interactions between HCV and melanoma were explored in Ingenuity Pathway Analysis (IPA) software version 01-22-01. IPA is a comprehensive repository of meticulously curated biological connections and functional annotations yielded from extensive modeling, which involves millions of individual relationships among proteins, genes, complexes, cells/tissues, drugs, and diseases. The “Path Explorer” tool was employed to build up the shortest path between HCV and melanoma using literature-supported signaling pathways. “The Canonical Pathway” tool was used to overlay the curated metabolic and cell signaling pathways associated with the network based on the literature.

For step 2, the expression signatures of RNA sequencing experiments in the Gene Expression Omnibus for (a) melanoma versus normal skin (GSE100050, GSE122907, GSE4587, and GSE114445), (b) nonalcoholic fatty liver disease versus HCV steatosis (E-MTAB-6863), and (c) hepatocellular carcinoma (HCC) with HCV versus non-HCV (GSE82177) were compared using the Comparison Analysis tool. Canonical pathways were compared to the curated signaling pathways identified in step 1. Intersected pathways and common molecular targets in the HCV-melanoma network were selected.

For step 3, the transcriptomic signatures of melanoma experiments (GSE100050, GSE122907, GSE4587, and GSE114445) were overlayed onto the HCV-melanoma network using the “Analysis, Datasets and Lists” tools. Deregulated molecular targets were filtered for further investigation. The “Molecular Activity Predictor” (MAP) tool was then applied to predict the downstream effect of activation and inhibition of the deregulated molecules detected in the datasets.

For step 4, the “Pattern Search” tool was applied to find the match and anti-match public data driving HCV-related melanoma. The pattern of up- and downregulated genes in the final hierarchical network generated in step 3 was compared and scored to thousands of OmicSoft datasets. The significance (*p*-value of overlap) was calculated by Fisher’s exact test. A mismatch analysis with a z score cutoff of ≤2 was exported and analyzed to predict the effect of novel therapeutic modalities that can counteract the regulator effects network induced in HCV-related melanoma.

## 3. Results

### 3.1. Retrospective Cohort Analysis

#### 3.1.1. Characteristics of the Study Participants

**Figure 1:**
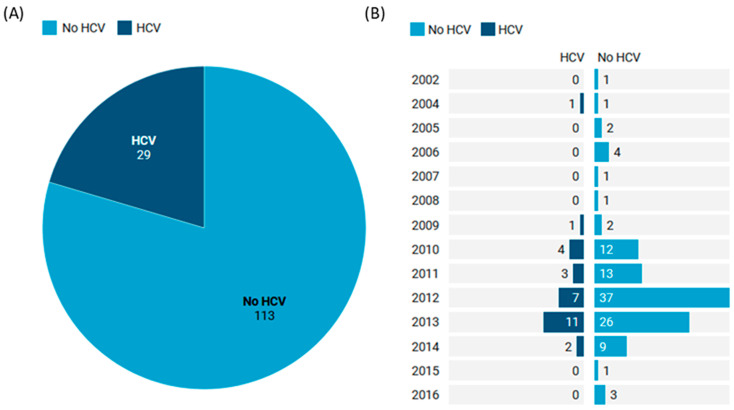
Distribution of melanoma patients. (**A**) Frequency of patients with HCV. (**B**) Distribution of samples according to the year of diagnosis.

The current study included 142 patients with cutaneous melanoma. Their mean age was 61.7 ± 14.9, and 77 patients (54.2%) were males. Their body mass index was 26.7 ± 5.39 kg/m^2^. About 21 cases (14.8%) had diabetes, 25 (17.6%) had hypertension, and 29 (20.4%) had HCV-induced cirrhosis (Table 1). The tumors were resected and collected between 2002 and 2016 (Figure 1).

The study included de novo tumors without pre-existing nevi lesions. The most affected sites were the extremities (45.1%), followed by the trunk (40.1%). Nodular melanoma was the most frequent type, accounting for 76.1% of the samples, while acral lentiginous melanoma was found in 17.6% of cases. A total of 18 patients (12.7%) were presented with multiple lesions (Table 1). As depicted in Table 2, most cases were clinical stage II (53.5%) and III (33.1%). Nodal infiltration was evident in 40.1% of cases, and 11.3% presented with distant metastasis. A total of 54.9% of cases had a *BRAF*^V600E^ gene mutation.

#### 3.1.2. Comparison between Patients with and without HCV-Induced Cirrhosis

There was no significant difference between HCV and non-HCV melanoma patients regarding their demographic features and comorbidities (Table 1). Both had a similar anatomical distribution of melanoma lesions (*p* = 0.18). However, there was a higher frequency of acral lentiginous melanoma in HCV cirrhotic patients compared to their counterparts (41.4% vs. 11.5%) and less representation of nodular melanoma (51.7% vs. 82.3%), *p* = 0.002 (Table 1).

According to the findings presented in Table 2, no notable differences were observed in the pathological features between patients with chronic hepatitis C and those without. In addition, the frequency of *BRAF*^V600E^ mutation was similar between the two cohorts (55.2% in HCV vs. 54.9% in non-HCV, *p* = 0.97).

All patients underwent surgical resection of their lesions. Post-operative adjuvant therapy was common in the HCV group (*p* = 0.042), as expected. The most-used therapy was interferon (24.1% in HCV vs. 6.2% in non-HCV, *p* = 0.009). No BRAF-targeted therapy or immune checkpoint inhibitors were reported in the patients’ medical records. During patient follow-up, 33 patients (23.3%) experienced relapse, and 45.1% developed recurrence after a period of improvement (Table 3). Although patients with HCV had a higher frequency of lower Clark levels (levels I and II: 58.6% versus 31.9%, *p* = 0.06), they were more likely to develop early relapse (41.4% versus 18.6%, *p* = 0.014) and recurrence (65.5% versus 39.8%, *p* = 0.020) (Table 2 and Table 3).

#### 3.1.3. Survival Analysis

**Figure 2:**
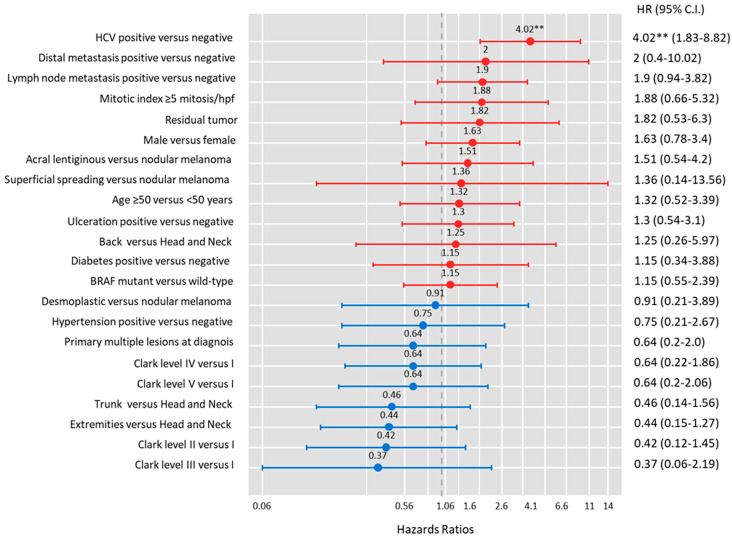
Multivariate Cox regression model for overall survival. The outcome was mortality, and other factors were included in the model as independent predictor factors. Hazards ratio and 95% confidence interval (CI) are reported. ** *p* < 0.001.

Of the 142 cohorts, 45 (31.7%) died after a median period of 112 months. Cutaneous melanoma patients with concomitant HCV infection had a higher risk of mortality (65.5% with HCV versus 23.0% with non-HCV, *p* < 0.001) (Table 3). To identify the impact of HCV on overall survival and define other independent risk factors for mortality, we performed a multivariate Cox regression analysis. The model included demographic data, comorbidities, tumor location and type, tumor stage, and molecular data. Our results revealed that HCV comorbidities were associated with a four-times-higher risk of mortality (HR = 4.02, 95%CI = 1.83–8.82, *p* < 0.001) (Figure 2).

**Figure 3:**
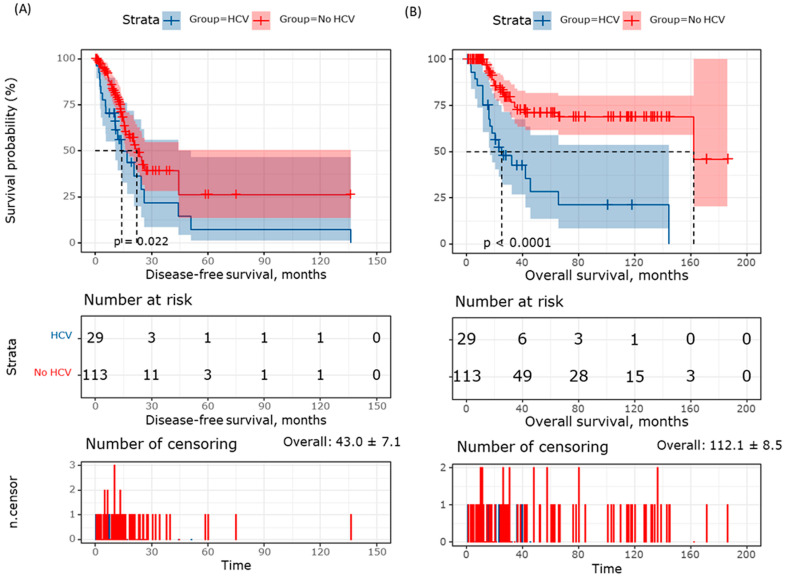
Kaplan–Meier survival curve. (**A**) Disease-free survival. (**B**) Overall survival. Survival times were assessed in months. The log-rank test was used to compare HCV and non-HCV patients.

Consistently, Kaplan–Meier curve analysis showed a lower survival probability for HCV patients and an early recurrence and mortality (Figure 3). The overall disease-free survival time was 43.0 ± 7.1 months. The DFS of the HCV group was 26.5 ± 8.7 months compared to the non-HCV group (50.4 ± 9.1 months) (*p* = 0.022). Similarly, the total overall survival time was 112.1 ± 8.5 months. The OS in the HCV cohort was 25.5 ± 8.4 months versus non-HCV patients (130 ± 9.2) (*p* < 0.001).

### 3.2. External Validation Using TriNetX Database

#### 3.2.1. Study Population

**Figure 4:**
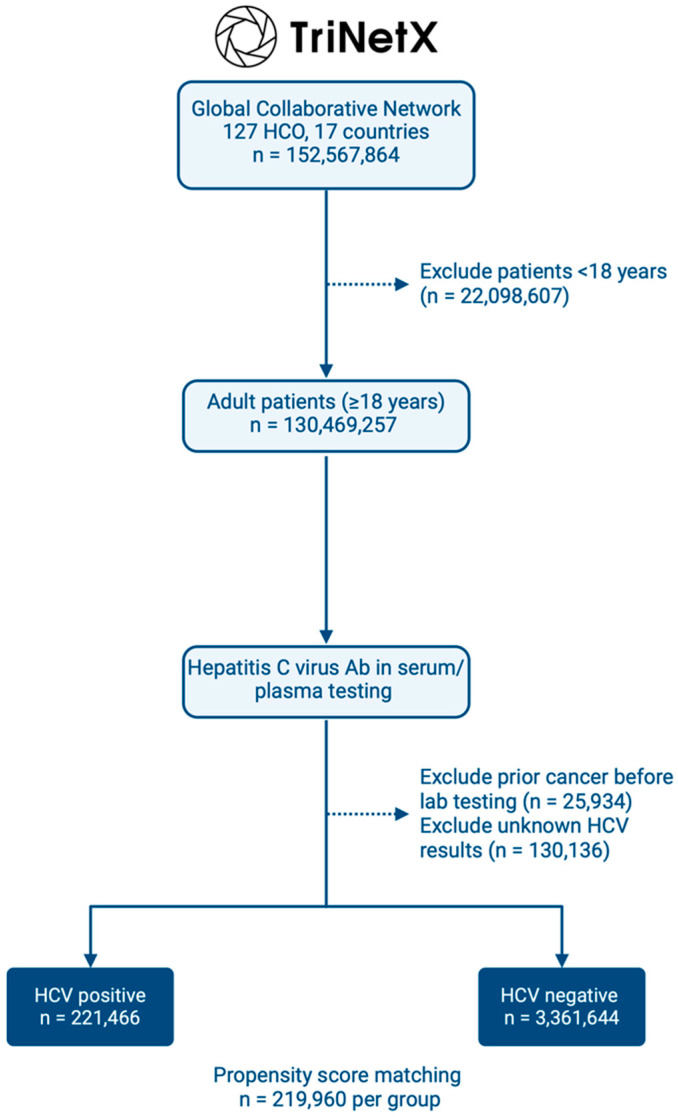
Workflow for patient recruitment from the TriNetX Global Collaborative Network.

The study utilized data from the TriNetX Global Collaborative Network, which included 127 healthcare organizations across 17 countries as of 1 August 2024. From a total of 152,566,936 patients, 130,469,257 adult patients were identified. After applying the inclusion and exclusion criteria, including an HCV antibody test and excluding those with prior cancer, the final cohort consisted of 221,466 HCV-positive patients and 3,361,644 HCV-negative patients (Figure 4).

Propensity score matching was then performed to balance the two groups, resulting in 219,960 patients in each group. Before matching, significant differences were observed between the HCV-positive and HCV-negative groups regarding demographics and comorbidities. HCV-positive patients were older (mean age 49.9 vs. 46.9 years, *p* < 0.001), more likely to be male (51.1% vs. 41.4%, *p* < 0.001), and less likely to be white (34.3% vs. 57.6%, *p* < 0.001) or Hispanic/Latino (4.5% vs. 9.7%, *p* < 0.001). HCV-positive patients also had higher rates of alcohol-related disorders (6.9% vs. 3.2%, *p* < 0.001) but lower rates of obesity, diabetes, hypertension, and metabolic disorders (all *p* < 0.001). After propensity matching, these differences were no longer statistically significant, with *p*-values >0.05 for all variables, indicating a successful balancing of the two groups (Table 4).

#### 3.2.2. Risk of Melanoma in HCV Patients

The HCV+ cohort had a longer mean follow-up of 43.9 ± 50.6 months versus 33.3 ± 34.7 months for the HCV- cohort, with median follow-up times of 24.4 months (IQR 63.7) and 22.1 months (IQR 49.6), respectively. During the follow-up period, the HCV+ group experienced 1637 melanoma cases (0.7%), while the HCV- group had 3485 cases (1.6%). The HCV+ patients showed a 53% lower risk of developing melanoma than the HCV- patients (risk ratio of 0.470, 95% CI: 0.443–0.498, *p* < 0.001).

#### 3.2.3. Outcomes of Melanoma Patients

**Figure 5:**
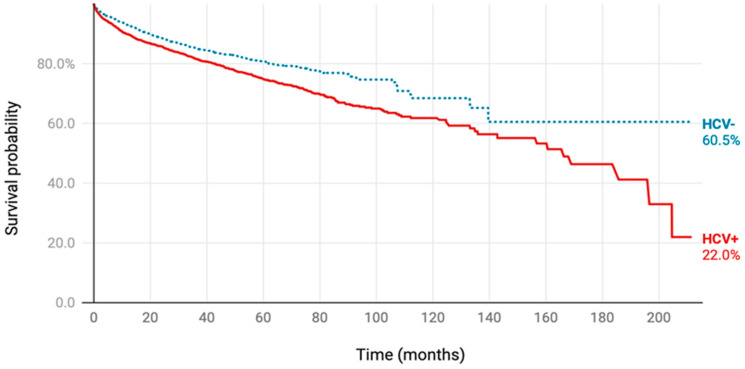
Overall survival in melanoma patients with and without HCV infection. Kaplan–Meier curve is shown, and the log-rank test was used.

A total of 2576 HCV+ melanoma patients were initially compared to 109,413 HCV-melanoma patients. After propensity matching, 2556 patients per group were analyzed. The mean follow-up was 38.4 ± 36.9 months for HCV+ melanoma patients and 36.8 ± 31.3 months for HCV- melanoma patients, with median follow-ups of 27.5 and 29.3 months, respectively. Regarding recurrence after six months, 692 (27.1%) of the HCV+ melanoma patients experienced recurrence compared to 754 (29.5%) of the HCV- melanoma patients (*p* = 0.054), with a hazard ratio of 0.912 (95% CI: 0.822–1.011). For all-cause mortality, HCV+ melanoma patients had a significantly higher risk (n = 512, 20.0%) compared to HCV- melanoma patients (n = 367, 14.4%), with a hazard ratio of 1.360 (95% CI: 1.189–1.556, *p* < 0.001). A Kaplan–Meier analysis with the log-rank test confirmed a significant difference in overall survival between the two groups (*p* < 0.001), as shown in Figure 5.

### 3.3. In Silico Data Analysis

#### Generating Regulatory Network Connecting HCV and Melanoma

**Figure 6:**
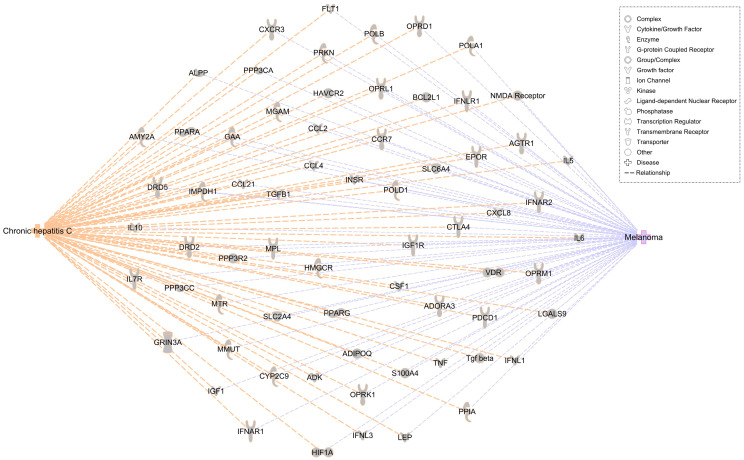
Identifying connecting molecular targets between HCV and cutaneous melanoma. The network was generated using Ingenuity Pathway analysis based on the findings in the QIAGEN Knowledge Base.

Based on prior publications, 183 and 13,249 associated molecules were identified for HCV and melanoma, respectively, in the IPA repository. The two disease nodes were connected using the “Path Explorer” tool. There were 67 shortest pathways, with the directionality connecting HCV to melanoma (Figure 6). The network generated was overlayed, and the top canonical pathways were identified. Table 5 displays the canonical pathways in which molecules within the network were found to participate.

**Figure 7:**
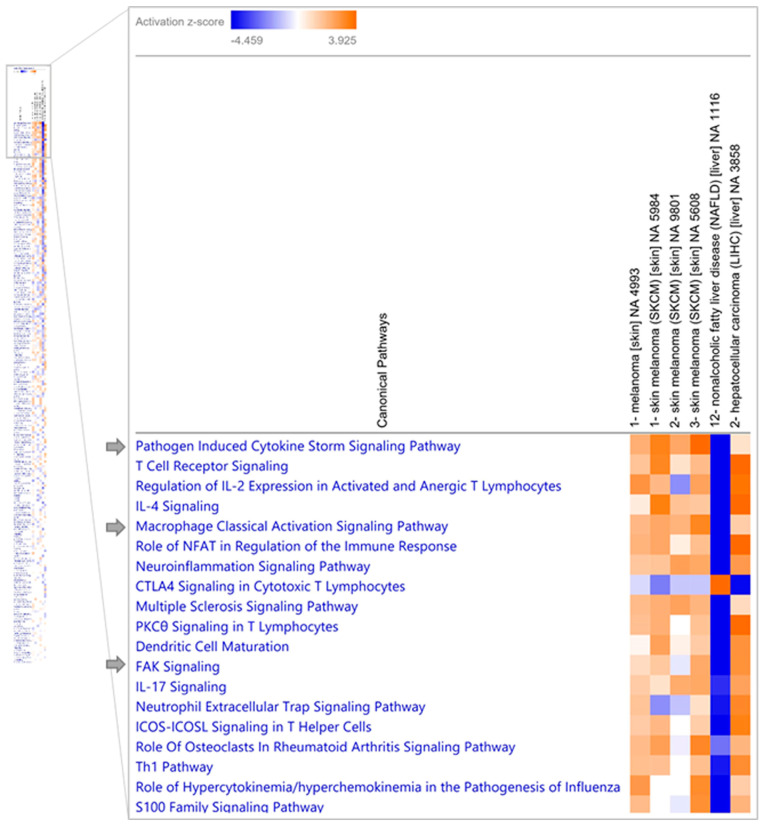
Comparison analysis of RNA-sequencing experiments for melanoma versus normal skin and HCV versus non-HCV. The pathway heatmap displays the z-scores from pathway activity analysis (orange and blue rectangles) for activated and inhibited signaling pathways. From left to right are the following experiments: GSE100050, GSE122907, GSE4587, GSE114445, E-MTAB-6863, and GSE82177.

Next, we performed a comparative analysis for the transcriptomic signature in (a) melanoma versus normal skin samples (GSE100050, GSE122907, GSE4587, and GSE114445), (b) nonalcoholic liver versus HCV (E-MTAB-6863), and (c) HCV+ HCC versus HCV- (GSE82177) (Figure 7). We found three pathways that intersected with the canonical pathways associated with our HCV-melanoma network, namely (1) the pathogen-induced cytokine storm signaling pathway, (2) the macrophage classical activation signaling pathway, and (3) the focal adhesion kinase (FAK) signaling. These three pathways were activated in both melanoma and HCV infection.

**Figure 8:**
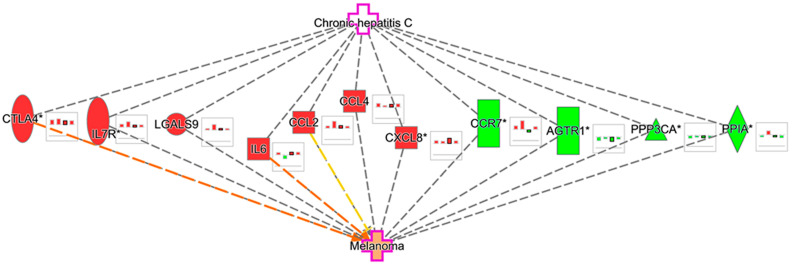
Overlay of melanoma experiments on HCV-melanoma network. The highlighted molecular nodes were deregulated in four melanoma experiments (left to right bars: GSE100050, GSE122907, GSE4587, and GSE114445). Red node: upregulated, green node: downregulated, orange node: predicted activation, orange line = leads to activation, yellow line = inconsistent findings. * Indicates that the gene/protein in the related dataset file has duplicates mapped to a single gene/protein in the network.

The transcriptomic signatures of the four melanoma experiments (GSE100050, GSE122907, GSE4587, and GSE114445) were overlayed on our generated HCV-melanoma network. Only deregulated molecular targets were kept. MAP prediction confirmed the effect of selected markers, especially IL6 and CTLA4, on the activation of melanoma disease (Figure 8).

An expression-pattern search revealed 522 mismatched conditions (z score <−2). Of these, 31 datasets represented comparisons between treatment versus control or treatment1 versus treatment2 in skin tissue. Table 6 displays the putative drugs that significantly lead to a reverse expression signature in skin diseases.

## 4. Discussion

Patients with chronic HCV and consequent hepatic cirrhosis present a potentially vulnerable population. In our study, we found melanoma patients with HCV were at increased risk of poorer outcomes regarding early relapse/recurrence and decreased survival than patients without HCV infection. Also, a multivariate Cox regression analysis supported this result, as HCV comorbidities were associated with four times more mortality risk. These findings are consistent with the results of others who reported that HCV seropositivity/infection is associated with an expanded group of cancers other than hepatocellular carcinomas, such as B-cell non-Hodgkin’s lymphomas, cholangiocarcinoma, lung, pancreas, esophagus, and thyroid, among others, that could increase the risk of mortality [5,12,31].

While the exact molecular mechanisms underlying the genetic association between HCV and melanoma are poorly understood, several hypotheses could be proposed. One theory could suggest that HCV may induce oxidative stress and a chronic systemic inflammatory state, leading to DNA damage and the potential for melanoma development [13,32]. Another could speculate on the direct implication of HCV in the skin precancerous lesion or pathogen-induced cytokine storm HCV-specific T cells, altering the gene expression and leading to uncontrolled growth and proliferation [33,34]. Further research is needed to explore these mechanisms and improve our understanding of the molecular link between HCV and melanoma.

On performing functional annotations by IPA and comparative analysis for the transcriptomic signature of both study entities, we found that three pathways, namely the “Pathogen induced cytokine storm”, “Macrophage classical activation”, and “FAK signaling”, were activated in both melanoma and HCV infection, in which two key players, IL6 and CTLA4, were essentially activated and had significant implications in the curated HCV-melanoma network.

HCV infects hepatocytes and liver macrophages to upregulate the production of pro-inflammatory cytokines and chemokines. For example, the Kupffer cells exposed to HCV-infected hepatocytes upregulated interleukin (IL)-1B expression, a well-known pro-inflammatory marker [35]. Though inflammation is natural and a protective physiological response to infection, chronic inflammation can induce fibrosis, cirrhosis, and HCC [36]. In the context of HCV, IL-6 has been shown to play a significant role in the viral persistence/progression of liver fibrosis [37]. Elevated IL-6 levels have been associated with increased viral replication and impaired antiviral immune responses [38,39]. Furthermore, IL-6 has been implicated in tumor progression, angiogenesis, and immunosuppression [40,41]. A mutant human *IL-6* gene (c.370G > A translating to p.E124K [somatic missense]) was observed with melanoma in humans (COSMIC: observed in 2 of 78 samples) [42]. Homozygous mutant mouse IL-6 gene (knockout) decreases the size of a melanoma that involves transgenic human c-RET protein in mouse skin [43]. Also, it was reported to promote the survival and growth of malignant melanoma cells while inhibiting anti-tumor immune responses [44], which is consistent with the findings as one of the poor prognostic biomarkers in patients with metastatic melanoma [45] and those who received immune checkpoint inhibition in multi-variable analyses of the randomized trials [46]. The intricate relationship between IL-6, HCV, and melanoma highlights the need for further exploration and understanding of this cytokine’s multifaceted functions. Additionally, targeting IL-6 or its signaling could open avenues for improving the immune responses to melanoma. Several IL-6 inhibitors are currently in clinical trials for various malignancies [47], and our findings suggest that they may hold promise for patients with HCV-associated melanoma as well.

Concerning CTLA4, it is an essential immune regulator that mediates both negative co-stimulation signals to T cells and regulatory T (Treg)-cell extrinsic control of effector responses [48]. HCV has been shown to exploit the CTLA4 pathway to establish persistent infection [49]. Viral proteins interact with CTLA4, inhibiting T-cell activation and dampening the antiviral immune response. This hijacking of CTLA4 signaling by HCV highlights its crucial role in viral persistence and provides a potential target for therapeutic intervention [50]. On the other hand, in melanoma, CTLA4 plays a complex role in modulating tumor immunity. Overexpression of CTLA4 on regulatory T cells (Tregs) within the tumor micro-environment impairs the function of effector T cells, leading to immune tolerance and tumor progression [51]. Targeting CTLA4 with “immune checkpoint inhibitors”, such as ipilimumab, an antibody acting on human CTLA4 protein, has emerged as an exciting approach in melanoma treatment, particularly for progressing unresectable cutaneous melanoma in adult humans [52,53,54]. These inhibitors unleash the immune system to mount a robust anti-tumor response against melanoma cells, improving patient outcomes significantly [52,54].

The lower incidence of melanoma in individuals with HCV infection, yet a poorer prognosis for those who develop the disease, presents a complex and intriguing paradox that warrants a thorough investigation into the underlying mechanisms and associations. One possible explanation could be the immunomodulatory effects of HCV infection on the immune response against melanoma cells. The altered immune microenvironment in individuals with HCV may influence the development and progression of melanoma, leading to differences in incidence and prognosis [55]. Also, the intricate interactions between the HCV virus and the host’s immune system may shape the tumor microenvironment and tumor immune responses [56], contributing to the observed disparities in melanoma outcomes. Furthermore, dysregulation of the specific biological pathways associated with HCV infection and melanoma development, such as inflammation, oxidative stress, and cellular proliferation, could underlie the paradoxical relationship between incidence and prognosis [57]. Elucidating the molecular mechanisms and signaling pathways involved in these processes may shed light on the observed disparities in disease outcomes.

It is worth noting that cancer patients with concurrent HCV remain a challenge for oncologists, specifically those receiving chemotherapy. Conceivably, there lies a delicate balance in HCV reactivation due to immunosuppression and therapy targeting the malignancy itself. Mahale et al. reported that 36% (8/22) of cancer patients receiving chemotherapy had HCV reactivation [58]. Moreover, HCV-related hepatitis flare is not uncommon in those patients, with reports ranging from 10% to 43% [59].

This study has some limitations that warrant consideration. The hospital-based cohort was relatively small, collected from a single institution, though this type of cancer is relatively uncommon in our region. Using a retrospective cohort from a specific database may introduce selection bias, with certain patient characteristics potentially over/under-represented. We addressed this by implementing rigorous inclusion/exclusion criteria and propensity score matching. Data accuracy is another crucial limitation, as retrospective studies often rely on existing records that may be incomplete or inaccurate. We mitigated this by thoroughly validating the data collection process and utilizing multiple data sources when possible. Additionally, we have expanded our discussion to address the limitations of our data source, including the inability to provide exact patient-level overlaps for all parameters. We believe this approach provides valuable insights into the relationships between different parameters while maintaining the integrity of our data source and analysis. Despite these efforts, we recommend caution in extrapolating these results to broader populations without further evidence from prospective studies. More extensive multicenter prospective studies encompassing diverse ethnicities are warranted. Furthermore, the identified potential molecular players shared between melanoma and HCV should be validated by mechanistic studies to prove their potential as targets for individualized medicine.

### 4.1. Clinical Implications

The study findings highlight the need for a modified clinical approach to HCV-positive melanoma patients, incorporating enhanced screening, multidisciplinary collaboration, and careful patient management throughout the treatment process. Given the increased risk of poorer outcomes in HCV-positive melanoma patients, we recommend that healthcare providers consider enhanced screening protocols for this population. Regular dermatological assessments may be warranted for individuals with a history of HCV infection, particularly those with additional risk factors for melanoma. This could facilitate the early detection of melanoma, which is crucial for improving patient prognosis. Also, a multidisciplinary approach to treating those patients is vital. Collaboration among oncologists, hepatologists, and infectious disease specialists could improve the management of care, particularly considering the potential complications from both melanoma and chronic HCV. This will ensure that antiviral therapy for HCV is considered in parallel with cancer treatment, aiming to minimize HCV-related complications during melanoma therapy.

Additionally, due to the higher risk of poorer outcomes, close monitoring of HCV-positive melanoma patients during treatment is essential. This includes assessing treatment tolerability, managing potential HCV reactivation, and monitoring for immune-related adverse events associated with immunotherapy. Regular follow-up to adapt treatment plans based on the patient’s response and emerging complications is crucial.

Regarding the suggested roles of IL-6 and CTLA4 in these patients, immunotherapy may need to be approached with caution. While immune checkpoint inhibitors have shown promise in melanoma treatment, the immunomodulatory effects of HCV infection could influence patient responses. Clinical trials designed to assess the safety and efficacy of combining antiviral agents with standard oncological treatments could provide crucial insights into optimizing patient outcomes.

### 4.2. Future Perspectives

A deeper investigation into the underlying processes that link HCV infection, melanoma incidence, and prognosis is essential to unravel the complexity of this paradox and inform potential interventions or targeted therapies/strategies for improving melanoma outcomes in individuals with HCV. Further studies exploring the immune responses, molecular pathways, and clinical characteristics associated with HCV and melanoma are warranted to elucidate the observed paradox’s mechanisms and clinical implications. Additionally, the exploration of targeted therapies, focusing on IL-6 and CTLA4, as well as investigating the immune microenvironment in HCV-infected melanoma patients, could lay a solid foundation for future therapeutic strategies.

Prioritizing research that utilizes genomic profiling, mechanistic studies, longitudinal tracking, experimental therapeutics, bioinformatics analyses, and population-based epidemiological studies to explore the genetic interactions between melanoma and HCV is highly recommended. By addressing these critical research aspects, we can better understand the underlying mechanisms and ultimately improve the management and outcomes for patients affected by both conditions.

## 5. Conclusions

In conclusion, HCV infection may have implications beyond liver disease, including an increased risk of developing skin conditions like melanoma. This connection highlights the importance of considering a patient’s HCV status when diagnosing skin conditions and treating patients with melanoma. Overall, healthcare practitioners should utilize a comprehensive approach when treating patients with HCV and be aware of the potential for related skin conditions like melanoma.

## Figures and Tables

**Table 1:** Demographic and clinical characteristics of the study population.

Variables		Total (*n* = 142)	No HCV (*n* = 113)	HCV (*n* = 29)	*p*-Value
Demographics					
Age at diagnosis, years	Mean ± SD	61.7 ± 14.9	61.5 ± 14.5	62.6 ± 16.5	0.73
	<50 years	34 (23.9)	26 (23)	8 (27.6)	0.63
	≥50 years	108 (76.1)	87 (77)	21 (72.4)	
Sex	Female	65 (45.8)	52 (46)	13 (44.8)	0.91
	Male	77 (54.2)	61 (54)	16 (55.2)	
Body mass index, kg/m^2^	Mean ± SD	26.7 ± 5.39	26.8 ± 5.52	25.7 ± 4.61	0.43
Obesity	Negative	73 (74.5)	60 (72.3)	13 (86.7)	0.34
	Positive	25 (25.5)	23 (27.7)	2 (13.3)	
Diabetes mellitus	Negative	121 (85.2)	99 (87.6)	22 (75.9)	0.14
	Positive	21 (14.8)	14 (12.4)	7 (24.1)	
Hypertension	Negative	117 (82.4)	94 (83.2)	23 (79.3)	0.59
	Positive	25 (17.6)	19 (16.8)	6 (20.7)	
Clinical data					
Origin	Pre-existing nevi	0 (0.0)	0 (0.0)	0 (0.0)	NA
	De novo	142 (100)	113(100)	29 (100)	
Anatomic location	Head and neck	12 (8.5)	11 (9.7)	1 (3.4)	0.18
	Extremities	64 (45.1)	46 (40.7)	18 (62.1)	
	Back	9 (6.3)	7 (6.2)	2 (6.9)	
	Trunk	57 (40.1)	49 (43.4)	8 (27.6)	
Multiple lesions at presentation	Negative	124 (87.3)	99 (87.6)	25 (86.2)	0.76
	Positive	18 (12.7)	14 (12.4)	4 (13.8)	
Subtype	Superficial spreading	3 (2.1)	2 (1.8)	1 (3.4)	**0.002**
	Acral lentiginous	25 (17.6)	13 (11.5)	12 (41.4)	
	Nodular melanoma	108 (76.1)	93 (82.3)	15 (51.7)	
	Desmoplastic	6 (4.2)	5 (4.4)	1 (3.4)	

Data are presented as frequency (percentage) or mean ± standard deviation. Fisher’s exact and student *t*-tests were used. The bold value indicates statistical significance at *p* < 0.05. NA: Not applicable.

**Table 2:** Pathological characteristics of malignant melanoma samples.

Variables		Total (*n* = 142)	No HCV (*n* = 113)	HCV (*n* = 29)	*p*-Value
Clinical stage	I	3 (2.1)	1 (0.9)	2 (6.9)	0.17
	II	76 (53.5)	62 (54.9)	14 (48.3)	
	III	47 (33.1)	36 (31.9)	11 (37.9)	
	IV	16 (11.3)	14 (12.4)	2 (6.9)	
T stage	T1	1 (0.7)	0 (0)	1 (3.4)	0.23
	T2	7 (4.9)	5 (4.4)	2 (6.9)	
	T3	16 (11.3)	13 (11.5)	3 (10.3)	
	T4	118 (83.1)	95 (84.1)	23 (79.3)	
N stage	Negative	85 (59.9)	69 (61.1)	16 (55.2)	0.67
	Positive	57 (40.1)	44 (38.9)	13 (44.8)	
M stage	M0	126 (88.7)	99 (87.6)	27 (93.1)	0.53
	M1	16 (11.3)	14 (12.4)	2 (6.9)	
Ulceration	Negative	49 (34.5)	38 (33.6)	11 (37.9)	0.67
	Positive	93 (65.5)	75 (66.4)	18 (62.1)	
Mitotic rate	<5 mitosis/hpf	107 (75.4)	88 (77.9)	19 (65.5)	0.23
	≥5 mitosis/hpf	35 (24.6)	25 (22.1)	10 (34.5)	
Breslow depth, mm	Median (IQR)	12.0 (7.0–17.7)	7.0 (3.0–11.5)	10.0 (1.5–19)	0.32
Clark level	I	24 (16.9)	16 (14.2)	8 (27.6)	0.06
	II	29 (20.4)	20 (17.7)	9 (31)	
	III	13 (9.2)	13 (11.5)	0 (0)	
	IV	39 (27.5)	32 (28.3)	7 (24.1)	
	V	37 (26.1)	32 (28.3)	5 (17.2)	
*BRAF* ^V600E^	Wild type	64 (45.1)	51 (45.1)	13 (44.8)	0.97
	Mutant	78 (54.9)	62 (54.9)	16 (55.2)	

Data are presented as frequency (percentage) or median (interquartile range). Fisher’s exact and Mann–Whitney U tests were used. Statistical significance at *p* < 0.05.

**Table 3:** Management and outcomes in malignant melanoma patients.

Variables		Total (*n* = 142)	No HCV (*n* = 113)	HCV (*n* = 29)	*p*-Value
Treatment					
Surgical margin	Free	130 (91.5)	106 (93.8)	24 (82.8)	0.07
	Residue	12 (8.5)	7 (6.2)	5 (17.2)	
Adjuvant therapy	Negative	113 (79.6)	94 (83.2)	19 (65.5)	**0.042**
	Positive	29 (20.4)	19 (16.8)	10 (34.5)	
Type of post-surgical adjuvant therapy	Radiotherapy	1 (0.7)	1 (0.9)	0 (0)	0.61
	DTC	5 (3.5)	3 (2.7)	2 (6.9)	0.27
	IFN	14 (9.9)	7 (6.2)	7 (24.1)	**0.009**
	CVD	8 (5.6)	6 (5.3)	2 (6.9)	0.66
	Dacarbazine	1 (0.7)	0 (0)	1 (3.4)	0.20
	Sunitinib	2 (1.4)	2 (1.8)	0 (0)	0.47
Follow-up					
Relapse	Negative	109 (76.8)	92 (81.4)	17 (58.6)	**0.014**
	Positive	33 (23.2)	21 (18.6)	12 (41.4)	
Recurrence/Progression	Negative	78 (54.9)	68 (60.2)	10 (34.5)	**0.020**
	Positive	64 (45.1)	45 (39.8)	19 (65.5)	
Died	Negative	97 (68.3)	87 (77.0)	10 (34.5)	**<0.001**
	Positive	45 (31.7)	26 (23.0)	19 (65.5)	

Data are presented as frequency (percentage) or mean ± standard deviation. Fisher’s exact and Mann–Whitney U tests were used. Bold values indicate statistical significance at *p* < 0.05. DTC: Dopachrome tautomerase, IFN: interferon, CVD: polychemotherapy regimen (cisplatin, vinblastine, and dacarbazine).

**Table 4:** Characteristics of matched cohort before and after matching.

Variables	Before Propensity Matching			After Propensity Matching		
	HCV+ (*n* = 221,466)	HCV− (*n* = 3,361,644)	*p*-Value	HCV+ (*n* = 219,960)	HCV− (*n* = 219,960)	*p*-Value
Demographics						
Age at Index	49.9 ± 17.2	46.9 ± 17.6	<0.001	49.9 ± 17.2	49.6 ± 17.0	0.46
Sex						
Female	106,181 (48.3%)	1,946,281 (58.2%)	<0.001	106,181 (48.3%)	106,558 (48.4%)	0.26
Male	112,452 (51.1%)	1,385,082 (41.4%)		112,435 (51.1%)	112,212 (51%)	
Race						
White	75,390 (34.3%)	1,926,769 (57.6%)	<0.001	75,390 (34.3%)	75,747 (34.4%)	0.26
Black	26,338 (12%)	445,344 (13.3%)		26,338 (12%)	26,503 (12%)	
Asian	2130 (1%)	143,202 (4.3%)		2130 (1%)	2101 (1%)	
NH/PI	117 (0.1%)	7191 (0.2%)		117 (0.1%)	121 (0.1%)	
AI/AN	537 (0.2%)	12,043 (0.4%)		537 (0.2%)	514 (0.2%)	
Other Race	6458 (2.9%)	271,374 (8.1%)		6458 (2.9%)	6420 (2.9%)	
Ethnicity						
Not Hispanic or Latino	93,880 (42.7%)	244,4574 (73.1%)	<0.001	93,880 (42.7%)	94,072 (42.8%)	0.56
Hispanic or Latino	10,005 (4.5%)	325,829 (9.7%)		10,005 (4.5%)	9689 (4.4%)	
Comorbidities						
BMI30–39 kg/m^2^	3904 (1.8%)	151,328 (4.5%)	<0.001	3904 (1.8%)	3699 (1.7%)	0.18
BMI ≥ 40 kg/m^2^	1813 (0.8%)	77,020 (2.3%)	<0.001	1813 (0.8%)	1703 (0.8%)	0.06
Nicotine dependence	8218 (3.7%)	159,883 (4.8%)	<0.001	8218 (3.7%)	8540 (3.8%)	0.17
Alcohol-related disorders	15,176 (6.9%)	106,752 (3.2%)	<0.001	15,176 (6.9%)	15,267 (6.9%)	0.89
Diabetes mellitus	19,142 (8.7%)	364,746 (10.9%)	<0.001	19,142 (8.7%)	19,535 (8.9%)	0.36
Hypertensive diseases	40,085 (18.2%)	825,475 (24.7%)	<0.001	40,085 (18.2%)	40,903 (18.6%)	0.11
Metabolic disorders	38,716 (17.6%)	968,894 (29%)	<0.001	38,716 (17.8%)	39,577 (18%)	0.13

Data are presented as mean ± standard deviation for continuous variables and n (%) for categorical variables. Comparisons between groups were performed using two-sided independent *t*-tests for continuous variables and chi-square tests for categorical variables. A *p*-value < 0.05 was considered statistically significant. Propensity score matching was performed using the nearest neighbor algorithm with a caliper width of 0.2 standard deviations of the logit of the propensity score. BMI: body mass index; AI/AN: American Indian or Alaska Native; NH/PI: Native Hawaiian or Other Pacific Islander.

**Table 5:** Canonical pathways associated with hepatitis C virus infection and melanoma network.

Canonical Pathway	Molecules	Targets
Hepatic fibrosis/hepatic stellate cell activation	18	AGTR1, CCL2, CCL21, CCR7, CSF1, CXCR3, FLT1, IFN, IFNAR1, IFNAR2, IGF1, IGF1R, IL6, IL10, LEP, TGFB1, TNF
FAK signaling	17	ADORA3, AGTR1, CCR7, DRD2, DRD5, FLT1, IFN, IFNAR1, INFLR1, IGF1R, IL7R, OPRD1, OPRK1, OPRL1, OPRM1, TGFB1, TNF
Pathogen-induced cytokine storm signaling pathway	16	CCL2, CCL4, CCL21, CXCL8, CXCR3, HMGCR, IFN, IFNA2, IFNAR1, IFNAR2, IL5, IL6, IL10, LEP, SLC2A4, TGFB1, TNF
Tumor microenvironment pathway	14	CCL2, CSF1, CTLA4, CCL8, HIF1A, IGF1, IL6, IL10, LEP, LGALS9, SLC2A4, TGFB1, TNF
Macrophage classical activation signaling pathway	12	CXCL8, HIF1A, IFN, IFNA2, IFNAR1, IFNAR2, IL5, IL6, IL10, LEP, PPARG, TGFB1, TNF
T-cell exhaustion signaling pathway	9	CTLA4, HAVCR2, IFN, IFNAR1, IFNAR2, IL6, IL10, LGALS9, PDCD1, TGFB1
HIF-alpha signaling	9	EPO, FLT1, HIF1A, IGF1, PPP3CA, PPP3CC, PPP3R2, SLC2A4, TGFB1
IL-12 signaling production in macrophages	7	CXCR3, IFN, IFNA2, PPAERG, TGFB1, TNF, VDR
Th1 and Th2 activation pathway	7	CXCR3, HAVCR2, IFN, IFNAR1, IL5, LGALS9, TGFB1
Role of cytokines in mediating communication between immune cells	6	IFN, IFNA2, IFNL1, IL5, TGFB1, TNF

**Table 6:** Pattern search results to find experiments with mismatched findings for HCV-related melanoma networks.

Disease State	GEO ID	Treatment	*p*-Value	Overlap Ratio	Overlap
Alopecia areata	GSE45551	Etanercept TNFi	7.4 × 10^−10^	6/11 (54.5%)	[AGTR1, CCL2, CCL4, CCR7, CTLA4, IL7R]
Atopic dermatitis	GSE133477	Crisaborole	4.24 × 10^−14^	8/11 (72.7%)	[AGTR1, CCL2, CCL4, CCR7, CTLA4, CXCL8, IL6, IL7R]
	GSE140684	Ustekinumab	7.8 × 10^−8^	5/11 (45.5%)	[AGTR1, CCR7, CTLA4, CXCL8, IL6]
	GSE32473	Betamethasone	8.12 × 10^−8^	5/11 (45.5%)	[AGTR1, CCL2, CCR7, CTLA4, CXCL8]
	GSE58558	Cyclosporine	4.07 × 10^−14^	8/11 (72.7%)	[AGTR1, CCL2, CCL4, CCR7, CTLA4, CXCL8, IL6, IL7R]
Normal control	GSE52360	Diphenylcyclopropenone	9.47 × 10^−10^	6/11 (54.5%)	[AGTR1, CCL2, CCR7, CXCL8, IL6, IL7R]
Psoriasis	GSE41663	Betamethasone	7.92 × 10^−8^	5/11 (45.5%)	[AGTR1, CCL4, CXCL8, IL6, IL7R]
	GSE85034	Methotrexate	9.87 × 10^−10^	6/11 (54.5%)	[AGTR1, CCL2, CCR7, CTLA4, CXCL8, IL7R]
		Adalimumab	8.54 × 10^−8^	5/11 (45.5%)	[CCL2, CCR7, CTLA4, CXCL8, IL7R]
Psoriasis Vulgaris	GSE106992	Ustekinumab	5.04 × 10^−6^	4/11 (36.4%)	[AGTR1, CTLA4, CXCL8, IL7R]
	GSE117239	Ustekinumab	7.96 × 10^−8^	5/11 (45.5%)	[AGTR1, CCR7, CTLA4, CXCL8, IL7R]
	GSE117468	Ustekinumab	8.12 × 10^−8^	5/11 (45.5%)	[AGTR1, CCL2, CTLA4, CXCL8, IL7R]
		Brodalumab	5.02 × 10^6^	4/11 (36.4%)	[AGTR1, CTLA4, CXCL8, IL7R]
	GSE136757	PF06700841	7.8 × 10^−8^	5/11 (45.5%)	[CCL4, CTLA4, CXCL8, IL6, IL7R]
Skin melanoma	GSE64741	UACC62	5.16 × 10^−6^	4/11 (36.4%)	[AGTR1, CTLA4, CXCL8, IL7R]
Systemic scleroderma	GSE100212	Bleomycin	6.72 × 10^−8^	5/11 (45.5%)	[CCL2, CCL4, CCR7, CTLA4, IL6]

## Data Availability

The original contributions presented in the study are included in the article/Supplementary Material, further inquiries can be directed to the corresponding authors.

## Supplementary Materials

The following supporting information can be downloaded at https://www.mdpi.com/article/10.3390/medicina60091531/s1: Table S1: Diagnostic and classification codes were utilized in the study.
