# Supplementary material for: The HCV-Melanoma Paradox: First Multi-Cohort and Molecular Net-Work Analysis Reveals Lower Incidence but Worse Outcomes—Integrating Clinical, Real-World, and In Silico Data
Source: Medicina (Kaunas). 2024 Sep 19;60(9):1531. doi: 10.3390/medicina60091531 (PMC11433761; doi:10.3390/medicina60091531)
Supplement: Supplementary file 1 [file medicina-60-01531-s001.zip › medicina-3167018-supplementary.pdf]

Table S1. Diagnostic and classification codes were utilized in the study.

Criteria	Category	Code	Description
**Inclusion criteria**			
HCV status	laboratory	UMLS: LNC:13955-0	Hepatitis C virus Ab in Serum or Plasma by Immunoassay (labResult: Positive or Negative)
**Exclusion criteria**			
Prior cancer	Globaloncology	UMLS: ICDO3:8000/3	Neoplasm, malignant
	Diagnosis	UMLS: ICD10CM:C80.1	Malignant (primary) neoplasm, unspecified
**Outcomes**			
Melanoma	Diagnosis	UMLS: ICD10CM:C43-C44	Melanoma and other malignant neoplasms of skin
	Diagnosis	UMLS: ICD10CM:D03	Melanoma in situ
	Diagnosis	UMLS: ICD10CM:C43	Malignant melanoma of skin
All-cause mortality	Demographics	Deceased	Deceased
	Diagnosis	UMLS: ICD10CM:R99-R99	Ill-defined and unknown cause of mortality (R99)
